# First Plastome Sequences of Two Endemic Taxa of *Orbea* Haw. from the Arabian Peninsula: Comparative Genomics and Phylogenetic Relationships Within the Tribe Ceropegieae (Asclepiadoideae, Apocynaceae)

**DOI:** 10.3390/biology15030223

**Published:** 2026-01-25

**Authors:** Samah A. Alharbi

**Affiliations:** Department of Biology, Faculty of Science, Umm Al-Qura University, Makkah 24381, Saudi Arabia; sarehaily@uqu.edu.sa

**Keywords:** *Orbea*, Stapeliinae, chloroplast genome, plastome evolution, arabian peninsula flora, fayfa mountains, genome rearrangement, phylogenomics, flip–flop inversion

## Abstract

*Orbea* is a morphologically diverse lineage within the subtribe Stapeliinae, yet plastome evolution in Arabian taxa remains insufficiently characterized. This study reports the first complete chloroplast genomes of *Orbea sprengeri* subsp. *commutata* and *O. wissmannii* var. *eremastrum* and investigates plastome structure, sequence variability, and phylogenetic relationships across tribe Ceropegieae. Chloroplast genomes were assembled, annotated, and compared with 13 published plastomes representing major Ceropegieae lineages. Both Arabian plastomes displayed the typical quadripartite structure and identical gene content of 114 unique genes, including 80 protein-coding genes, 30 transfer RNA genes, and four ribosomal RNA genes. However, *O. wissmannii* var. *eremastrum* exhibited pronounced structural divergence, possessing the largest plastome recorded for the tribe (170,054 bp), an 8.9 kb expansion of the inverted repeat regions, and an 8.4 kb inversion spanning the *ndhG–ndhF* region. Comparative analyses revealed conserved gene order across Ceropegieae but identified six highly variable loci (*accD*, *clpP*, *ndhF*, *ycf1*, *psbM–trnD*, and *rpl32–trnL*) as potential DNA barcodes. Selection pressure analyses indicated strong purifying selection across most genes, with localized adaptive signals in *accD*, *ndhE*, *ycf1*, and *ycf2*. Phylogenomic reconstruction consistently resolved the two Arabian *Orbea* taxa as a distinct clade separate from the African *O. variegata*. This study fills a gap in Ceropegieae plastid genomics and underscores the importance of sequencing additional *Orbea* species to capture the full extent of genomic variation within this diverse genus.

## Simple Summary

*Orbea* Haw. is a genus of succulent plants that grow in extremely dry regions, including parts of the Arabian Peninsula. These plants contribute to the unique biodiversity of desert ecosystems, yet their chloroplast genetic information has remained largely unstudied. This research presents the first complete chloroplast DNA sequences of two *Orbea* plants endemic to the Arabian Peninsula. By examining and comparing their genetic sequences with those of related species, several notable findings were revealed. One species possesses an unusually large chloroplast genome and a distinctive rearrangement of several genes, indicating a more complex evolutionary history than previously recognized. Regions of the DNA that show high levels of variation between species were also identified; these areas can support accurate plant identification and further studies on plant evolution. In addition, the relationship between the *Orbea* species was clarified, showing that the Arabian plants form a separate group from the African species. These results provide essential new information for understanding, conserving, and documenting the diversity of desert plants in the Arabian Peninsula.

## 1. Introduction

The tribe Ceropegieae is the third largest of the five tribes within the subfamily Asclepiadoideae (Apocynaceae), comprising a diverse group of c. 800 plant species distributed throughout the Old World tropics and subtropics [1,2]. It is divided into four subtribes—Anisotominae, Heterostemminae, Leptadeniinae, and Stapeliinae—each reflecting a distinct evolutionary lineage [3,4,5]. The stem-succulent stapeliads of the Stapeliinae form the core of the tribe Ceropegieae, encompassing c. 357 species in 31 genera [6] characterized by leafless, fleshy stems adapted to arid environments [7,8,9]. Among these, the genus *Orbea* Haw. is one of the largest, with about 60 recognized species [10] distributed from southern and eastern Africa to the Arabian Peninsula [2,11]. *Orbea variegata* (L.) Haw. (Syn. *Stapelia variegata* L), the type species, is among the most popular stapeliads, attracting many succulent enthusiasts for its striking appearance [12].

The southern regions of the Arabian Peninsula represent a center of succulent diversity, harboring numerous endemic species that contribute to the region’s status as a biodiversity hotspot [13,14]. Among these, species of the genus *Orbea* play a prominent role, with twelve taxa reported so far from the Arabian Peninsula (Table 1) [10,11,15,16,17]. The taxonomy of Arabian *Orbea* has undergone several revisions over the years, mirroring broader taxonomic shifts within the genus. The subsequent overview highlights key taxonomic developments in *Orbea*, focusing especially on revisions pertaining to the Arabian taxa.

The genus *Orbea* was originally described by Adrian Hardy Haworth in 1812 but was soon synonymized under *Stapelia* [11,18]. Until its resurrection by Leach in 1975, most species remained classified within either *Stapelia* or *Caralluma* [19,20,21,22,23,24,25]. Subsequently, Leach [26] further reviewed the genus and proposed segregating several groups into newly established genera, namely *Orbeopsis*, *Pachycymbium*, and *Orbeanthus*. Later, Gilbert [27] expanded *Pachycymbium* to include *Stultitia araysiana* from Yemen and other members of the ‘Ango Group’ of *Caralluma*, which primarily includes species of *Caralluma* occurring mainly in tropical Africa and Arabia, north of the equator. In 1994, Plowes transferred these species to *Angolluma*, a genus originally established by R. Münster in 1990 [28].

Subsequently, Bruyns [11], Bruyns [29] merged all segregate genera (*Orbeanthus, Orbeopsis, Pachycymbium*, and *Angolluma*) back into an expanded *Orbea*, based on detailed phylogenetic analyses. While Bruyns dismissed *Angolluma*, Plowes continued to recognize it and, in 2007, described two new species from Yemen (*A. cucullata* and *A. fenestrate*) [30], which were later transferred to *Orbea* by Meve [31]. Additionally, *Orbea nardii* was recorded from Oman by Raffaelli, Mosti, and Tardelli in 2008 [32]. Recently, Bruyns, Klak and Hanáček [8] proposed reducing *Orbea* to a section within a broadly circumscribed *Ceropegia* L., based on molecular evidence. However, *Orbea* continues to be recognized as a distinct genus by other authorities, such as Endress, Meve, Middleton and Liede-Schumann [2] and Plant of the World Online POWO [10], who recognize 55 and 60 species, respectively. A detailed timeline of the historical taxonomic changes involving Arabian *Orbea* taxa is provided in Table S1.

*Orbea* species possess considerable cultural and economic importance, particularly due to their medicinal and nutritional uses across their distribution range [7,33,34]. *Orbea variegata* has been extensively investigated for its phytochemical composition, especially pregnane glycosides, which exhibit diverse biological activities, including antimicrobial, anti-inflammatory, antioxidant, and anti-carcinogenic effects, with recent studies highlighting its potential in skin cancer treatment [35,36,37]. *Orbea deflersiana* is used to treat burns, eczema, diabetes, and wounds, and demonstrates strong antioxidant, antibacterial, and antifungal activities [33,38,39]. Similarly, *O. wissmannii* var. *wissmannii* is traditionally used in Yemen to treat stomach ulcers, constipation, and food poisoning, and exhibits notable antioxidant and antimicrobial activity, particularly against *Escherichia coli* [33,40]. In addition to their medicinal value, several *Orbea* species contribute to food security. *Orbea wissmannii* var. *wissmannii*, locally known as “khusmaa,” is still consumed by communities in southern Yemen during periods of food scarcity [34], while *O. wissmannii* var. *eremastrum*, known as “Adhba Kalbah” in Saudi Arabia, and *O. luntii*, locally called “Re Eoeoon” in Oman, are also traditionally used as food sources [41].

Despite their cultural and medicinal importance, the conservation status of Arabian *Orbea* is of growing concern. *Orbea sprengeri* subsp. *commutata*, *O. wissmannii* var. *eremastrum*, and *O. deflersiana* have been identified as high-priority taxa for conservation in Saudi Arabia due to their restricted distribution and vulnerability to environmental pressures [42,43,44]. These species face threats of habitat degradation, environmental stresses [45], and soil erosion in fragile environments like the Shada Mountains [46]. Similarly, in Oman, *O. nardii* and *O. luntii* are also critically endangered, with a small population size. These species are at risk due to overgrazing, rapid infrastructure development, and road construction in their habitats, leading to their inclusion in Oman’s national red list [16]. Urgent conservation measures are needed to protect these species and preserve their genetic diversity.

**Figure 1:**
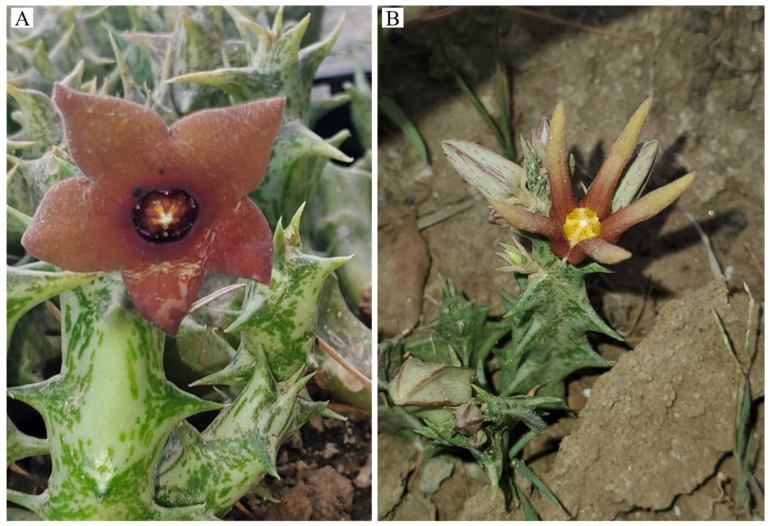
*Orbea sprengeri* subsp. *commutata* (**A**) and *Orbea wissmannii* var. *eremastrum* (**B**) in their natural habitat in Fayfa Mountain, southwestern Saudi Arabia. Photographs were taken by E. Elfaify and are reproduced here with permission.

Given the economic importance and threatened conservation status of *Orbea* species in the Arabian Peninsula, chloroplast (cp) genome sequencing provides a valuable framework for evolutionary and conservation-oriented investigations. Chloroplast genomes are particularly informative for phylogenetic and population studies due to their conserved quadripartite structure, relatively low recombination rates, and uniparental (typically maternal) inheritance, together with sufficient sequence variation to resolve relationships at inter- and intra-generic levels [47,48]. Recent plastome-based phylogenomic studies have demonstrated the effectiveness of cp genomes in resolving complex evolutionary histories, identifying maternal lineages, and clarifying taxonomic ambiguities, even in groups shaped by hybridization and polyploidy [49,50]. While plastid genomes represent a single genomic locus, their comparative analysis provides an essential foundation for evolutionary inference and conservation genomics, particularly when integrated with complementary nuclear and population-level data. For this study, *O. sprengeri* subsp. *commutata* and *O. wissmannii* var. *eremastrum* (Figure 1) were selected for plastome sequencing as they are endemic, endangered, and represent key taxa in the Arabian Peninsula’s succulent flora. Additionally, they face multiple conservation challenges, making them ideal candidates for genetic studies aimed at informing conservation strategies.

Currently, *O. variegata*, native to South Africa, is the only species in the genus with a complete chloroplast genome available in GenBank. To address the existing data gap for Arabian *Orbea* species, this study aimed to (1) assemble and annotate the complete chloroplast genomes of *O. sprengeri* subsp. *commutata* and *O. wissmannii* var. *eremastrum*; (2) characterize their genomic structure and sequence features; (3) conduct comparative analyses with available plastomes of tribe Ceropegieae; and (4) reconstruct their phylogenomic relationships within the tribe. This study will expand the genomic resources available for Arabian *Orbea* and provide a foundational framework for future genetic and conservation studies.

## 2. Materials and Methods

### 2.1. Plant Materials

Fresh and healthy stems of *O. sprengeri* subsp. *commutata* and *O. wissmannii* var. *eremastrum* were collected for this study in February 2023 by Mr. Essa El-Faify from the Fayfa Mountains, Saudi Arabia (17°15′51.6″ N, 43°06′44.4″ E, altitude 2200 m.a.s.l.). The plants were photographed at the collection site, and voucher specimens were preserved in 70% ethanol and stored in the spirit collection of the Umm Al-Qura University Herbarium, Department of Biology, Alzahir Campus, under accession numbers EFO3 and EFO4, respectively.

### 2.2. DNA Extraction, Library Construction, and Genome Sequencing

Dried stem fragments from the collected specimens were sent to Novogene Co., Ltd. (Beijing, China) for DNA extraction and sequencing. Total genomic DNA was extracted using the FastDNA™ SPIN Kit (MP Biomedicals, Irvine, CA, USA), following the manufacturer’s protocol. The purity and integrity of the extracted DNA were evaluated via agarose gel electrophoresis, while DNA concentration was measured using a Qubit Fluorometer (Thermo Fisher Scientific, Waltham, MA, USA).

For library preparation, genomic DNA was randomly sheared into fragments of ~350 bp. The fragments were subjected to end-repair, A-tailing, and ligation with Illumina adapters using the Rapid Plus DNA Lib Prep Kit for Illumina (ABclonal, Wuhan, China). Size selection, PCR amplification, and purification were performed with the AMPure XP system (Beckman Coulter, Beverly, MA, USA). The libraries were assessed for quality using the Agilent Fragment Analyzer System (Agilent Technologies, Santa Clara, CA, USA) and quantified using Qubit and qPCR. Sequencing was performed on an Illumina NovaSeq PE150 platform (Illumina, San Diego, CA, USA), generating 150 bp paired-end reads.

To ensure data quality, Fastp V.0.23.1 [51] was used to filter the raw reads. Quality control steps included (1) discarding reads with adapter contamination (>10 nucleotides aligned to the adapter with ≤10% mismatches); (2) removing reads with >10% ambiguous bases; and (3) filtering reads with >50% low-quality bases (Phred quality < 5). This process yielded 12 GB of high-quality data, constituting ~90% of the raw reads.

### 2.3. Genome Assembly and Annotation

Chloroplast genome assembly was conducted using NOVOPlasty v4.3.5 [52], with the *O. variegata* chloroplast genome (NC_079601.1) as a reference. Initial assemblies were refined by extracting the *rpoC2* and *ndhD* genes from *O. wissmannii* var. *eremastrum* and *O. sprengeri* subsp. *commutata*, respectively. These genes were subsequently used as seeds in a second round of assembly, with the “Extend seed directly” option enabled in the NOVOPlasty configuration file. This iterative approach successfully resulted in the complete circular chloroplast genomes for both taxa.

To verify assembly accuracy, independent de novo assemblies were also generated using GetOrganelle v1.7.7.1 [53]. Paired-end clean reads were assembled using the chloroplast genome mode with default parameters, employing a multi-k-mer strategy (−k 21, 45, 65, 85, 105) and 25 extension rounds (−R 25). The word size for read recruitment was set to 112 (−w 112). The published chloroplast genome of a closely related *Orbea* species was used as a reference for read recruitment. Assembly graphs were visualized using Bandage v0.8.1 [54].

The assembled genomes were annotated automatically using GeSeq v2.03 [55] and further refined manually in Geneious Prime^®^ v2025.0.3 [56]. Annotation validation was performed using GB2sequin v1.0 [57] and NCBI Genome Workbench v3.9.0. Circular genome maps were generated using OrganellarGenomeDRAW v1.3.1 [58]. The final annotated plastomes were submitted to GenBank under accession numbers PQ412530 and PQ412531 for *O. sprengeri* subsp. *commutata* and *O. wissmannii* var. *eremastrum*, respectively.

### 2.4. Comparative Analysis of Genomic Features

At the time of preparing this research, 16 chloroplast genomes from the tribe Ceropegieae were available in GenBank, representing 11 species from the subtribe Stapeliinae, one from Heterostemminae, three from Leptadeniinae, and one from Anisotominae (Table 2). However, three of these genomes were deposited as partial sequences, each lacking one copy of the inverted repeat (IR) region, and are highlighted in gray in Table 2. Consequently, only the complete plastomes were included in the comparative genomic analyses, while the partial sequences were used only for phylogenetic reconstruction. The genomes of *Duvalia velutina* (MT431578.1) and *Monolluma quadrangula* (MT413385.1) were annotated after being downloaded from GenBank using Geneious Prime.

Together with the two newly sequenced plastomes generated in this study, a total of 15 complete chloroplast genomes were included in the comparative analysis (Table 2). Genomic features, including length, gene content, intron/exon structures, and GC content, were obtained using Geneious Prime. Codon counts, Relative Synonymous Codon Usage (RSCU), and amino acid frequency analyses were performed using MEGA v11.0.13 [59]. Heatmaps depicting codon distribution for all protein-coding genes were generated using TBtools-II v2.136 [60]. The boundaries of the large single-copy (LSC), small single-copy (SSC), and IR regions were compared across the genomes using IR-scope [61].

### 2.5. Nucleotide Diversity and Sequence Divergence

Nucleotide diversity (Pi) was analyzed using two datasets: (1) the complete set of 15 Ceropegieae plastomes and (2) a subset including only the three *Orbea* taxa. For both datasets, whole chloroplast genome sequences were aligned using MUSCLE v3.8.4 [62] implemented in Geneious Prime. To minimize overestimation of nucleotide diversity resulting from the species-specific inversion in *O. wissmannii* var. *eremastrum*, the complementary strand of the inverted region was used in the alignment. Nucleotide diversity (Pi) was then calculated in DnaSP v6.12.03 [63] using a sliding window analysis with a window length of 800 bp and a step size of 200 bp. This window size represents a compromise between resolution and statistical stability [63] and has been commonly applied in plastome-wide diversity analyses to identify highly variable regions while reducing stochastic noise.

Sequence divergence and mutational hotspot regions among 15 Ceropegieae genomes were further investigated using mVISTA [64] under the Shuffle-LAGAN alignment mode, with the *O. variegata* plastome serving as the reference. The Shuffle-LAGAN algorithm is specifically designed for global alignment [65,66] of genomes and has been extensively used in comparative chloroplast genome analyses to visualize coding and non-coding divergence and to identify mutational hotspots in angiosperms.

### 2.6. Repeat Sequence Analysis

Dispersed repeats (forward, reverse, palindrome, and complement) were identified using REPuter [67]. Simple sequence repeats (SSRs) were detected with MISA v2.1 [68], using a minimal repeat threshold of 8, 5, 4, 3, 3, and 3 for mono-, di-, tri-, tetra-, penta-, and hexanucleotides, respectively. These thresholds were selected to balance detection sensitivity and reliability, follow the default and recommended settings of MISA [68,69], and are widely applied in chloroplast genome studies to reduce false positives associated with short or low-complexity repeats.

### 2.7. Selection Pressure Analysis

Selection pressure on 79 common protein-coding genes was assessed across 15 chloroplast genomes of the tribe Ceropegieae. The coding sequences (CDS) were aligned using Geneious Prime, and nonsynonymous (Ka) to synonymous (Ks) substitution rate ratios (Ka/Ks) were calculated in TBtools-II using the NG method and *O. variegata* (NC_079601.1) as the reference sequence. A Ka/Ks ratio greater than one indicates positive selection, a ratio equal to one suggests neutral evolution, and a ratio less than one reflects purifying selection.

### 2.8. Phylogenomic Analysis

Phylogenomic relationships were reconstructed using a dataset of 21 chloroplast genomes, comprising 18 plastomes from the tribe Ceropegieae (Table 2) and three outgroup species from the tribe Marsdenieae—*Hoya exilis* Schltr. (MW719054.1), *H. megalaster* Warb. ex K.Schum. & Lauterb. (MW719063.1), and *H. ariadna* Decne. (OL754671.1). Phylogenomic analysis was conducted using a concatenated dataset of 80 protein-coding genes. Each gene was individually aligned using MUSCLE within Geneious Prime, following default parameters. The aligned sequences were subsequently concatenated into a single dataset for tree reconstruction. Maximum likelihood (ML) and Bayesian inference (BI) phylogenies were constructed using RAxML v8.2.12 [70] with 1000 bootstrap replicates and MrBayes v3.2.7 [71], respectively, utilizing the CIPRES portal [72]. The best-fit evolutionary model (GTR+I+G) was determined using jModelTest2 v2.1.6 [73]. Convergence of BI analyses was assessed in Tracer v1.7.1, with effective sample sizes (ESS) >200 considered adequate. Tree visualization and annotation were performed in iTOL v6.5.8 [74].

## 3. Results

### 3.1. General Features of the Chloroplast Genomes

**Figure 2:**
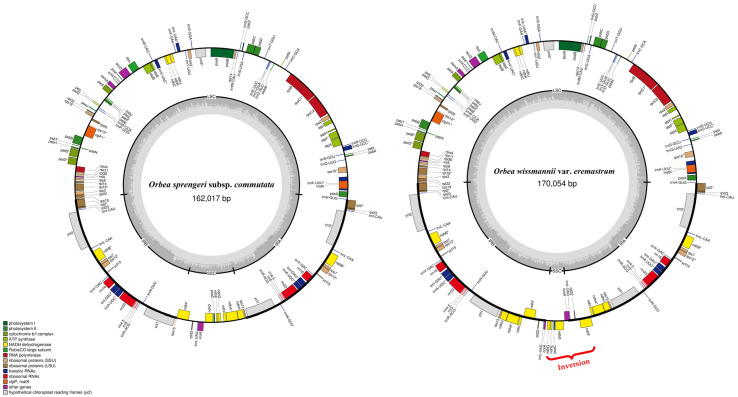
Plastid genome map of *Orbea sprengeri* subsp. *commutata* and *Orbea wissmannii* var. *eremastrum*. The inner circles, shaded in dark and light gray, represent the GC content. Functional gene groups are distinguished by various colors, with genes inside the circles transcribed in a clockwise direction, while those outside are transcribed in an anticlockwise direction. An asterisk (*) indicates genes containing introns.

The newly generated chloroplast genomes of *O. sprengeri* subsp. *commutata* and *O. wissmannii* var. *eremastrum* were fully assembled and annotated, with genome sizes of 162,017 bp and 170,054 bp, respectively. Both genomes displayed a typical quadripartite structure (Figure 2), including a large single-copy (LSC) region of 86,501 bp for *O. sprengeri* subsp. *commutata* and 85,882 bp for *O. wissmannii* var. *eremastrum*, a small single-copy (SSC) region of 13,213 bp and 4298 bp, and inverted repeat (IR) regions of 31,151 bp and 39,937 bp, respectively. Notably, *O. wissmannii* var. *eremastrum* exhibits a unique 8.4 kb bp flip-flop inversion spanning the region from *ndhG* to *ndhF*, along with a markedly 8.9 kb IR expansion into the SSC region (Figure 2). This IR expansion resulted in the duplication of several genes typically confined to the SSC region, including *rps15*, *rpl32*, *ndhA*, *ndhF*, *ndhH*, *ndhI*, and *trnL*-UAG. The combined effect of this inversion and IR boundary shift has led to gene relocations disrupting the organization of the *ndhH–ndhD* operon.

The chloroplast genome of *O. sprengeri* subsp. *commutata* encodes a total of 133 genes, comprising 88 protein-coding genes, 37 tRNA genes, and 8 rRNA genes (Table 3). In comparison, *O. wissmannii* var. *eremastrum* encodes 140 genes, including 94 protein-coding genes, 38 tRNA genes, and 8 rRNA genes (Table 3). The higher gene count in *O. wissmannii* var. *eremastrum* is primarily due to the expansion of the IR into the SSC region, resulting in the duplication of the aforementioned genes. Despite these differences in total gene number, both genomes contain the same number of unique genes—114 in total—comprising 80 protein-coding genes, 30 tRNA genes, and 4 rRNA genes.

The total length of coding sequences was 85,020 bp in *O. sprengeri* subsp. *commutata* and 90,876 bp in *O. wissmannii* var. *eremastrum*, corresponding to 52.4% and 53.1% of the genome, respectively. The remaining non-coding sequences include intergenic spacers and introns, accounting for 76,997 bp and 79,178 bp, respectively. The total GC content was nearly identical between the two species (37.7% in *O. sprengeri* subsp. *commutata* and 37.4% in *O. wissmannii* var. *eremastrum*), with the highest GC content consistently observed in the IR regions.

The chloroplast genomes of *O. sprengeri* subsp. *commutata* and *O. wissmannii* var. *eremastrum* each contain 17 unique genes with introns and exons, distributed across the LSC (12 genes), IR (4 genes), and SSC (1 gene) regions (Table 4). In *O. wissmannii* var. *eremastrum*, due to the expansion of the IR region to include *ndhA*, all intron-containing genes are located exclusively in the LSC and IR regions (Table 4). Among these, *ycf3*, *trnL-UAA*, and *ndhA* display consistent exon and intron lengths between the two taxa. The largest intron was observed in *trnK-UUU* (2527–2530 bp), located in the LSC region, while the longest exon was found in *rpoC1* (432 bp). Most genes contain a single intron, except for *ycf3* and *clpP1*, which contain two introns (Table 4).

The comparison of the newly assembled chloroplast genomes with 13 previously published plastomes from the tribe Ceropegieae reveals both conserved and divergent structural characteristics across its subtribes—Heterostemminae, Leptadeniinae, and Stapeliinae (Table 5). All taxa exhibit the typical quadripartite chloroplast structure consisting of an LSC, SSC, and a pair of IRs. Total genome sizes range from 158,487 bp in *Pentasachme caudatum* (Leptadeniinae) to 170,054 bp in *O. wissmannii* var. *eremastrum* (Stapeliinae). This variation is mainly due to differences in IR and SSC lengths. *Orbea wissmannii* var. *eremastrum* is especially notable for its significantly expanded IR region (39,937 bp) and a reduced SSC region (4298 bp), contrasting with other species, where SSC regions range from ~13,000 to over 20,000 bp.

Despite these structural variations, most taxa exhibit relatively consistent LSC lengths (~85,000–91,000 bp) and total GC content values (37.3–37.9%). Exceptions occur in the Leptadeniinae species *Leptadenia pyrotechnica* and *P. caudatum*, and Heterostemminae species *Heterostemma oblongifolium*, which display lower GC content in the LSC region (35.4% and 36%, respectively) and elevated IR GC content (43.2%). The proportion of coding sequences varies from 46% in *M. quadrangula* to 53.15% in *O. variegata*.

Gene counts are broadly conserved across subtribes, with most plastomes encoding 131–133 genes, including 83–88 protein-coding genes, 36–37 tRNA genes, and 8 rRNA genes. Again, *O. wissmannii* var. *eremastrum* stands out with the highest number of total genes (140) and protein-coding genes (94), largely due to gene duplications from IR expansion. Conversely, the Leptadeniinae species *P. caudatum* and *L. pyrotechnica* have the lowest gene counts (127 total; 83 protein-coding genes).

### 3.2. Codon Usage Analysis

**Figure 3:**
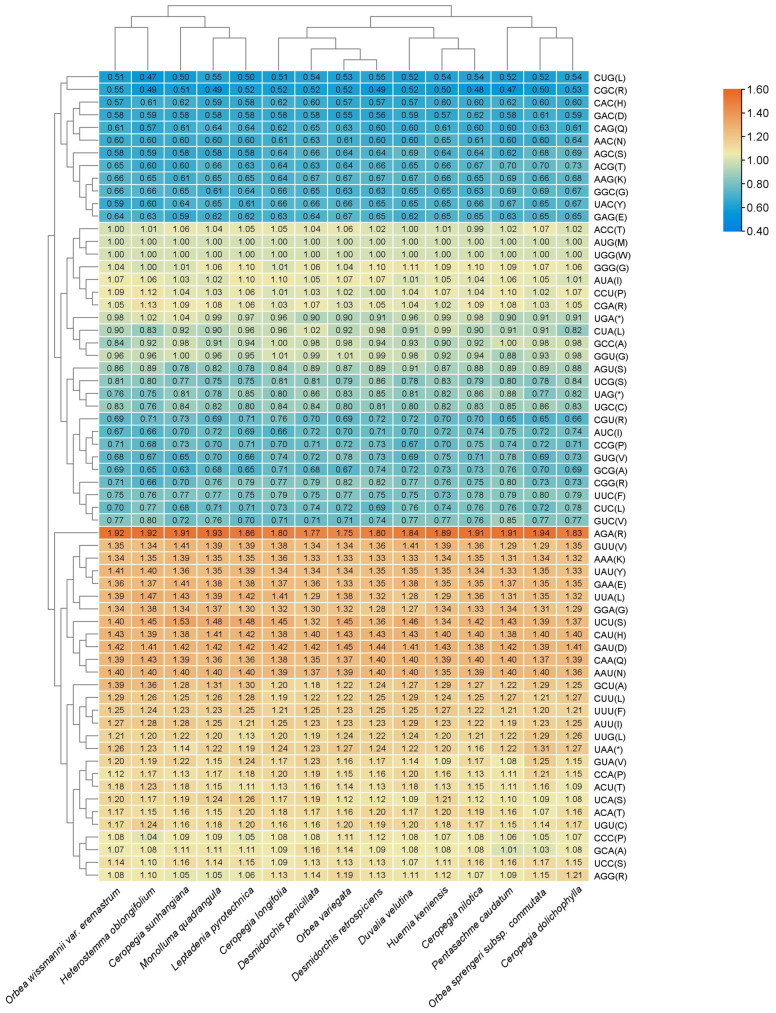
Heatmap analysis of codon distribution across all protein-coding genes for the species studied. Color key: Orange indicates higher RSCU values, while blue represents lower RSCU values. Stop codon marked with an asterisk (*).

The analysis of protein-coding genes and their relative synonymous codon usage (RSCU) values is shown in Table S2 and Figure 3. RSCU measures the frequency of synonymous codons encoding the same amino acid, identifying codon bias. Across all taxa, codons with RSCU > 1, considered preferred codons, predominantly end in A or U, indicating a strong A/T bias in the third codon position. For instance, codons such as UUA (leucine) and AUA (isoleucine) displayed consistently high RSCU values, reflecting a preference for these codons across multiple species. Conversely, codons with RSCU < 1, such as CGC (arginine) and GGC (glycine), were less frequently used, often ending in G or C. Codons with RSCU = 1, such as AUG (methionine) and UGG (tryptophan), showed no bias and were uniformly used in all plastomes.

On average, the protein-coding genes in the chloroplast genomes of Ceropegieae contained 52,829 to 57,035 codons, with slight variations among species. Among these, the codons encoding leucine were the most abundant. Stop codons showed clear preferences, with UAA being the most frequent across all plastomes, while UAG and UGA were less commonly utilized.

The newly sequenced chloroplast genomes of *O. sprengeri* subsp. *commutata* and *O. wissmannii* var. *eremastrum* demonstrated codon usage patterns consistent with other members of Stapeliinae. Heatmap visualization of RSCU values (Figure 3) revealed clustering of codon usage patterns among the taxa.

**Figure 4:**
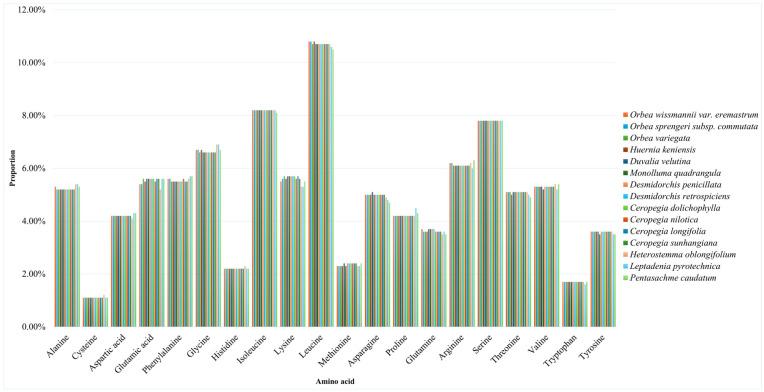
Amino acid frequencies in the chloroplast genome of tribe Ceropegieae species.

The analysis of amino acid frequencies across the chloroplast genomes of 15 taxa in the tribe Ceropegieae reveals consistent patterns with minor variations among species (Figure 4). Leucine was the most abundant amino acid in all plastomes, accounting for approximately 10.5–10.9% of the total codons. Other highly abundant amino acids include serine (approximately 7.6–8.0%) and isoleucine (approximately 8.2%). In contrast, the least frequent amino acids were tryptophan (approximately 1.7%) and cysteine (approximately 1.1%). Methionine, which serves as the start codon for nearly all protein-coding genes, was moderately represented at approximately 2.3–2.4%.

### 3.3. IR Expansion and Contraction

**Figure 5:**
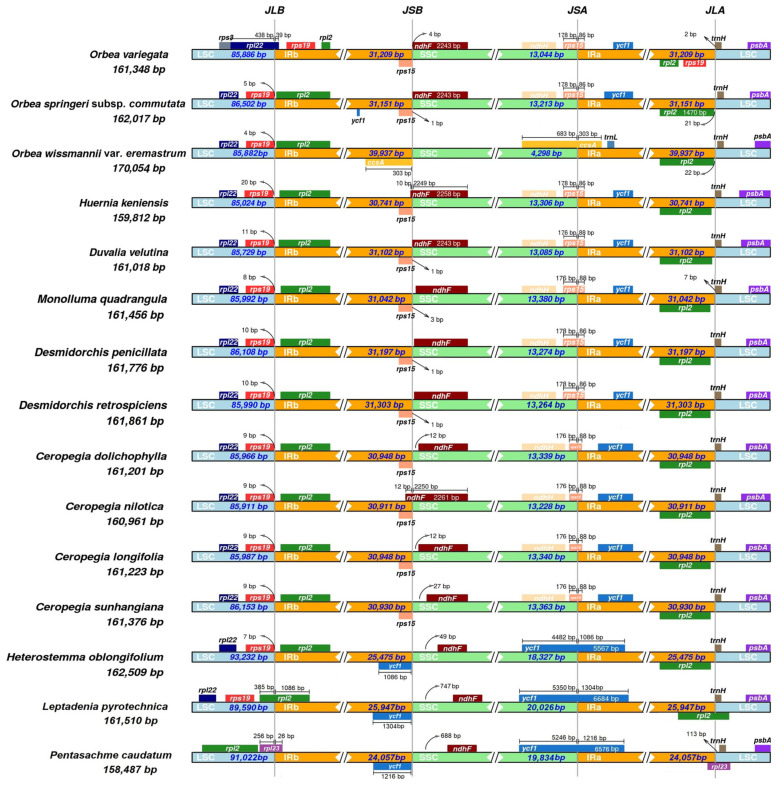
Comparison of inverted repeat (IR) boundaries among 15 chloroplast genomes of tribe Ceropegieae. The figure is schematic and not to scale. SSC, small single copy; LSC, large single copy.

The comparison of IR boundaries across the 15 Ceropegieae plastomes revealed considerable variation in the contraction and expansion of the IR regions, reflecting structural diversity among species (Figure 5). In the newly sequenced plastomes of Arabian *Orbea* taxa, the LSC/IRb junction lies between *rps19* and *rpl22*, while the LSC/IRa boundary is located between *rpl2* and *trnH-GUG*. Both genomes also exhibit IR expansion into the SSC region, though the extent differs significantly. In *O. sprengeri* subsp. *commutata*, the SSC/IRa junction spans *rps15*, whereas in *O. wissmannii* var. *eremastrum*, the expansion is more extensive, placing the junction within *ccsA* and resulting in the near-complete incorporation of the SSC into the IRs, leaving only ~4 kb of SSC remaining. Similarly, the SSC/IRb boundary in *O. sprengeri* subsp. *commutata* lies between *ndhF* and *rps15*, while in *O. wissmannii* var. *eremastrum*, it falls within *ccsA*.

Subtribe-level comparisons revealed distinct boundary patterns, especially at the SSC/IRa junction. Plastomes of Stapeliinae generally exhibit IR expansions into the SSC that span *rps15*, a pattern shared by all sampled members of this subtribe except *O. wissmannii* var. *eremastrum*, which shows a more extreme expansion. In contrast, species of Heterostemminae and Leptadeniinae have SSC/IRa junctions that span *ycf1*. These shifts in IR boundaries have resulted in the formation of pseudogenes: *rps15* pseudogenes in the IRb region of Stapeliinae species, and *ycf1* pseudogenes in the IRb region of Heterostemminae and Leptadeniinae.

At the IRb/LSC junction, the *rps19* gene is typically located within the LSC across most species, with minor positional variation. However, *O. variegata* displays IR expansion into the LSC, placing *rpl22* at the IRb/LSC border. Notably, *P. caudatum* (Leptadeniinae) is the only species in the dataset that exhibits IR contraction, resulting in *rpl23* spanning the IRb/LSC junction. Conversely, the LSC/IRa boundary is highly conserved across all species, consistently marked by the *trnH-GUG* gene situated entirely within the LSC region.

### 3.4. Identification of Variable Regions

**Figure 6:**
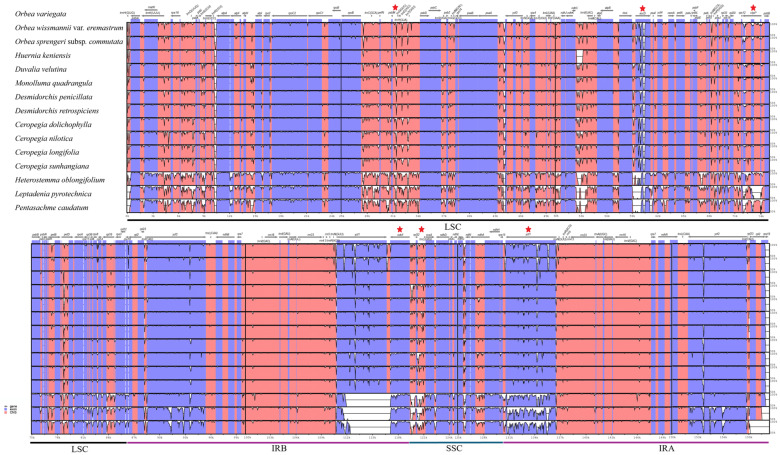
Comparative alignment of 15 Ceropegieae chloroplast genomes with *Orbea variegata* as the reference. The *y*-axis indicates the percentage identity (50–100%), and the *x*-axis represents genomic coordinates. Upper arrows denote the position and orientation of each gene. Red stars highlight hypervariable regions in the tribe identified by sliding-window nucleotide diversity analysis, including *psbM-trnD* (0.048), *accD* (0.018), *clpP* (0.047), *ndhF* (0.050), *rpl32-trnL* (0.048), and *ycf1* (0.024). CNS = conserved non-coding sequences.

Nucleotide diversity (Pi) was analyzed across two datasets: one including 15 Ceropegieae plastomes and another comprising only the three *Orbea* taxa. The Pi values, calculated across 800 bp windows, ranged from 0 to 0.0076 across the 15 plastomes and from 0 to 0.047 among the three *Orbea* taxa. In both datasets, the LSC region exhibited the highest sequence variability, while the IR regions were the most conserved (Figure S1). Among the protein-coding genes in the 15 plastomes, *clpP* (0.076) displayed the highest nucleotide diversity, followed by *accD* (0.073), *ycf1* (0.063), and *ndhF* (0.050) (marked with red stars in Figure 6), making these loci key contributors to overall plastome variability. In *Orbea* taxa, peaks of variability were likewise concentrated in coding regions, with the highest Pi values observed for *clpP* (0.047), *ycf1* (0.024), *accD* (0.018), and *ycf2* (0.015). While intergenic spacer (IGS) regions generally exhibited lower nucleotide variability, certain regions, such as *psbM-trnD* (0.048) and *rpl32-trnL* (0.048) (marked with red stars in Figure 6), showed moderate levels of diversity in the 15 plastomes.

The mVISTA-based pairwise comparison of the 15 Ceropegieae chloroplast genomes, using *O. variegata* as the reference (Figure 6), reveals detailed patterns of conservation and divergence across coding and non-coding regions. The LSC and SSC regions show greater sequence variability compared to the IRs, which remain highly conserved. Significant peaks of nucleotide diversity are observed in several IGS regions, including *psbA-trnH*, *ndhC-trnV*, *rpl32-trnL*, and *trnT*-*psbD*. Among the protein-coding genes, loci such as *ycf1*, *ycf2*, *clpP*, and *accD* display notable variability; genes associated with photosynthetic functions, such as *rbcL* and *psbA*, exhibit relatively high conservation.

### 3.5. Repeats Identification

**Figure 7:**
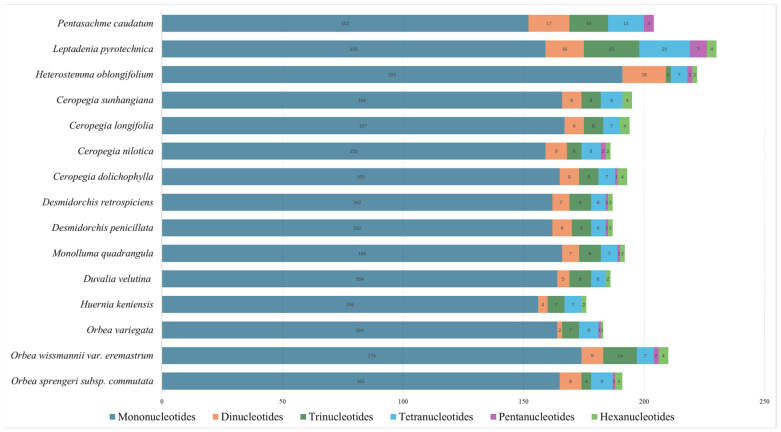
Number of SSR types in the chloroplast genomes of tribe Ceropegieae species.

The analysis of simple sequence repeats (SSRs) in the chloroplast genomes of Ceropegieae species revealed substantial variation in both the total number and types of SSRs, as well as their motif frequencies, across taxa (Figure 7 and Figure S2, Tables S3 and S4). Mononucleotide SSRs were the most abundant class, with counts ranging from 152 (*P. caudatum*) to 191 (*H. oblongifolium*), and were predominantly composed of A/T motifs, reflecting the AT-biased composition of chloroplast genomes. The newly sequenced *O. sprengeri* subsp. *commutata* and *O. wissmannii* var. *eremastrum* contained 165 and 174 mononucleotide repeats, respectively. Dinucleotide SSRs, primarily represented by AT/AT motifs, were less frequent, ranging from 2 to 18, while trinucleotide SSRs, dominated by AAG/CTT motifs, varied from 2 to 23. Tetranucleotide repeats ranged from 6 to 21, with motifs such as AAAT/ATTT being the most prominent, while pentanucleotide and hexanucleotide repeats were rare, generally occurring in frequencies of 1–7.

Among the taxa, *L. pyrotechnica* exhibited the highest overall SSR count (230) and diversity, including rarer repeats such as pentanucleotides and hexanucleotides. *Heterostemma oblongifolium* also showed high SSR diversity, particularly in tetranucleotides and pentanucleotides. In contrast, *Huernia keniensis* had the lowest SSR count (176). The SSR profiles of *O. sprengeri* subsp. *commutata* and *O. wissmannii* var. *eremastrum* were consistent with other members of Stapeliinae, highlighting the dominance of mononucleotide A/T motifs and limited representation of higher-order repeats.

The majority of SSRs are typically concentrated in the LSC region, followed by the SSC and then the IR regions across most taxa. This general pattern also holds true for *Orbea* species, except for *O. wissmannii* var. *eremastrum*, which exhibits a distinct deviation—showing an unusually high number of SSRs in both IR regions and a corresponding reduction in the SSC (Figure S3). This atypical distribution aligns with the pronounced IR expansion in *O. wissmannii* var. *eremastrum*, which has incorporated large portions of the SSC into the IRs, leading to the relocation of SSRs.

**Figure 8:**
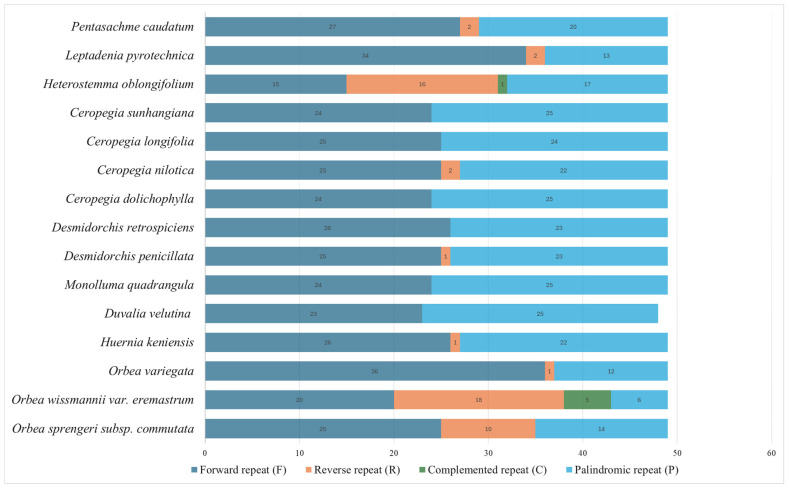
Number of dispersed repeats in the chloroplast genomes of tribe Ceropegieae species.

The analysis of dispersed repeats revealed notable variation in the number and types of repeats, which include forward (F), reverse (R), palindromic (P), and complemented (C) repeats (Figure 8, Table S5). Forward repeats were the most abundant, ranging from 15 (*H. oblongifolium*) to 36 (*O. variegata*), with the newly sequenced *O. sprengeri* subsp. *commutata* and *O. wissmannii* var. *eremastrum* containing 25 and 20 forward repeats, respectively. Palindromic repeats were also common, ranging from 6 (*O. wissmannii* var. *eremastrum*) to 25 (*C. dolichophylla*, *C. sunhangiana*, *Du. velutina*, and *M. quadrangula*). Reverse repeats were less frequent, with counts ranging from absent to 18, the highest being in *O. wissmannii* var. *eremastrum*, while complemented repeats were the least frequent, observed in only two species, *H. oblongifolium* (1) and *O. wissmannii* var. *eremastrum* (5).

Dispersed repeats were distributed across a combination of protein-coding genes, tRNA genes, and IGS regions (Figure S4, Table S5). Among the protein-coding genes, *ycf1* and *ycf2* consistently exhibited the highest repeat counts across most species. Other protein-coding genes, such as *petB*, *accD*, *petD*, *ndhA*, *rpl16*, and *rps15*, also harbored notable repeat counts, while loci like *psbB* and *infA* contained repeats in fewer species. Additionally, dispersed repeats were identified in tRNA genes, including *trnS-GCU*, *trnS-GGA*, and *trnK-UUU*, which were distributed across multiple species. Palindromic repeats were frequently found in protein-coding genes like *ycf1*, *ycf2*, and *accD*.

A comparative analysis between the two newly sequenced plastomes revealed notable differences in the abundance and distribution of dispersed repeats. *Orbea sprengeri* subsp. *commutata* exhibited a greater number of forward repeats (25), distributed across IGS regions (21) and several protein-coding genes, including *accD* (2), *petD* (1), and *ycf1* (1). Palindromic repeats were also more numerous (n = 14), all located in the IGS regions, while reverse repeats (n = 10) were likewise confined to IGS regions. No complement repeats were detected. In contrast, *O. wissmannii* var. *eremastrum* contained all four repeat types, including forward repeats, which were observed in both IGS (11) and *petB* (9), while palindromic (6), complemented (5), and reverse (18) repeats were limited to IGS. These patterns highlight species-specific repeat architectures, with *O. sprengeri* subsp. *commutata* showing broader distribution across both coding and non-coding regions, and *O. wissmannii* var. *eremastrum* displaying greater repeat type diversity, but with more localized distribution.

### 3.6. Selection Pressure

**Figure 9:**
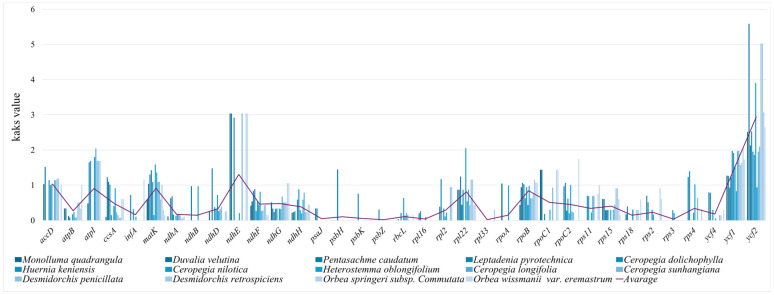
Ka/Ks ratio of 35 protein-coding genes across 15 chloroplast genomes of tribe Ceropegieae.

The Ka/Ks values of 79 unique protein-coding genes across 15 Ceropegieae plastomes were assessed to evaluate selection pressure (Table S6). After excluding genes with undefined or zero Ka/Ks values, 35 genes retained meaningful estimates (Figure 9). Of these, 31 genes exhibited Ka/Ks ratios below one, indicating that they were under purifying selection. Only four genes—*accD*, *ndhE, ycf1*, and *ycf2*—showed Ka/Ks values greater than one, suggesting that these genes may have experienced adaptive evolution. No gene exhibited a Ka/Ks ratio equal to one, indicating that none of the genes were evolving under neutral selection.

### 3.7. Phylogenomic Relationships

**Figure 10:**
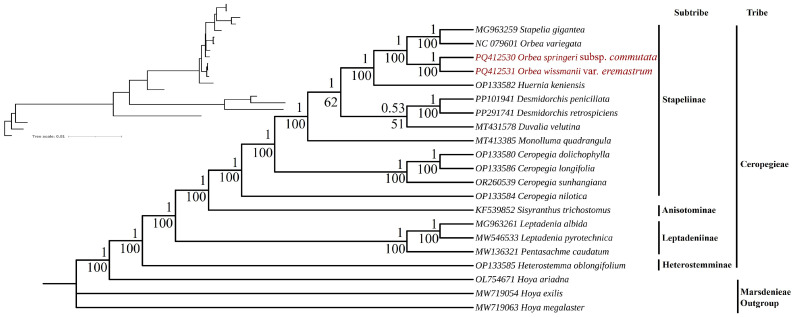
Maximum likelihood phylogenomic tree of 21 Ceropegieae species and outgroup taxa based on 80 chloroplast protein-coding genes. The upper-left inset displays the tree with branch lengths. Posterior probabilities (PP) are shown above the branches, and ML bootstrap values are annotated below. Taxa newly sequenced in this study are shown in red.

The phylogenomic relationships among 21 species of the tribe Ceropegieae and outgroup taxa were reconstructed based on 80 protein-coding genes using Bayesian inference (BI) and maximum likelihood (ML) methods. Both analyses produced identical topologies; as a result, only the ML tree is presented, showcasing well-supported branches with high bootstrap values and posterior probabilities (Figure 10). The tribe Ceropegieae was resolved as monophyletic, with distinct clades representing the subtribes Stapeliinae and Leptadeniinae.

Within Stapeliinae, the three *Orbea* taxa—*O. sprengeri* subsp. *commutate*, *O. wissmannii* var. *eremastrum*, and *O. variegata*—formed a well-supported subclade. However, *O. wissmannii* var. *eremastrum* and *O. sprengeri* subsp. *commutata* exhibiting closer evolutionary ties (posterior probabilities (PP) = 1; bootstrap (BP) = 100%), whereas *O. variegata* grouped more distantly within the same subclade alongside *Stapelia gigantea*. These species clustered within the broader Stapeliinae group, which included genera such as *Desmidorchis*, *Huernia*, *Duvalia*, and *Monolluma*. The genus *Ceropegia* formed a distinct group, appearing as a sister to stapeliad genera within the Stapeliinae. The subtribe Leptadeniinae, which included species such as *L. pyrotechnica*, *L. albida*, and *P. caudatum*, formed a distinct and well-supported clade. Anisotominae, represented by a single species, *Sisyranthus trichostomus*, was resolved as a sister lineage to the Stapeliinae clade. Heterostemminae, represented solely by *H. oblongifolium*, was positioned as the earliest diverging lineage within the tribe.

## 4. Discussion

The newly sequenced and annotated chloroplast genomes of *O. sprengeri* subsp. *commutata* and *O. wissmannii* var. *eremastrum* share a conserved quadripartite structure and identical gene content (114 unique genes), consistent with typical eudicots plastomes, including other members of Apocynaceae [48,75,76,77,78,79,80,81,82,83,84]. However, *O. wissmannii* var. *eremastrum* exhibits distinct plastome rearrangements, including an 8.4 kb inversion spanning *ndhG* to *ndhF* and an 8.9 kb expansion of the IR regions into the SSC. These rearrangements resulted in an increase in total plastome size, alterations in gene order, and duplication of several genes typically confined to the SSC, distinguishing it from *O. sprengeri* subsp. *commutata* and other Ceropegieae taxa.

In most higher plants, plastid DNA encodes eleven *ndh* genes [85,86] that produce NDH polypeptides forming the thylakoid NDH complex [87,88], which enables plants to withstand diverse terrestrial stresses and maintain photosynthetic efficiency [89]. This complex, functionally analogous to mitochondrial complex I, transfers electrons from NADH to plastoquinone [90]. Among these genes, *ndhC*, *ndhK*, and *ndhJ* are transcribed as a single operon (*ndhC–J*) within the LSC region [91], whereas *ndhH*, *ndhA*, *ndhI*, *ndhG*, *ndhE*, *psaC*, and *ndhD* form the *ndhH–D* operon in the SSC region [92]. The *ndhF* gene is also located in the SSC, and two identical copies of *ndhB* occur in the IRs (one per copy), likely transcribed independently as monocistronic mRNAs [90]. The NDH complex requires all eleven plastid-encoded subunits plus nucleus-derived components [89,93]; thus, inversions disrupting *ndh* operons could affect transcriptional coordination and complex assembly.

In *O. wissmannii* var. *eremastrum*, the 8.4 kb inversion disrupts the canonical *ndhH–D* operon (Figure 2), which may potentially influence transcriptional regulation and co-expression. Loss or pseudogenization of plastid *ndh* genes has occurred repeatedly and independently across diverse plant lineages. This pattern is most pronounced in heterotrophic taxa such as *Orobanche* and many Orchidaceae [94,95], but also in fully photosynthetic species including *Pinus thunbergii* [96], members of Ericaceae [97,98], *Gentiana* sect. *Kudoa* [99], some Cactaceae [100], *Corydalis* [101], and *Capparis* [102]. At present, the biological significance of the inversion observed in *O. wissmannii* var. *eremastrum* remains speculative, and it is unclear whether it represents a fixed structural feature or a rare plastome configuration. If validated, this inversion would represent the first documented case within Apocynaceae and could provide a useful system for investigating operon stability, transcriptional flexibility, and plastome evolution within the family. Targeted population-level sampling combined with long-read sequencing will be essential to determine whether this inversion reflects a stable plastome architecture, an isomeric variant, or possible plastome heterogeneity.

The total plastome sizes of *O. sprengeri* subsp. *commutata* and *O. wissmannii* var. *eremastrum* are 162,017 bp and 170,054 bp, respectively. Both fall within the range typically reported for Apocynaceae [75,103] and are comparable to the average plastome size in eudicots [104]. Variation in chloroplast genome size is generally attributed to differences in intergenic region length, expansion or contraction of IR regions, and differences in gene content [105]. In this study, the ~9 kb size difference between the two Arabian *Orbea* plastomes is mainly due to a large-scale expansion of the IR regions observed in *O. wissmannii* var. *eremastrum*, where each IR measures 39,937 bp. This represents a substantial increase over the typical IR size of 20–30 kb in most angiosperms [83]. In typical angiosperm plastomes, only a small portion (~1000 bp) of the *ycf1* gene is duplicated in the IR [106]. However, in *O. wissmannii* var. *eremastrum*, IR expansion extends well into the SSC region, resulting in the duplication of the full *ycf1*, *rps15*, *rpl32*, *ndhA*, *ndhF*, *ndhH*, *ndhI*, *trnL-UAG*, and a pseudogenized copy of *ccsA*. Such large-scale IR expansions have been documented in other Apocynaceae taxa, including *Alyxia sinensis* from subfamily Rauvolfioideae [75] and members of the Marsdenieae tribe (Asclepiadoideae) such as *Dischidia*, *Hoya*, and *Papuahoya* [107,108]. Similar events have also been reported in distantly related lineages, including *Plantago ovata* (Plantaginaceae) [109], *Asarum* (Aristolochiaceae) [110], and *Euphorbia* (Euphorbiaceae) [111], suggesting that IR boundary dynamics are recurrent and potentially lineage-specific features of chloroplast genome evolution. The traveling of the gene from SSC or LSC to IRs or vice versa affects the rate of mutations; mostly, the genes that travel from LSC or SSC to IRs showed a low rate of evolution [103].

In contrast, *O. sprengeri* subsp. *commutata* displays a more typical IR/SSC boundary pattern, with the junction extending into *rps15*. This configuration is consistent with other Stapeliinae plastomes such as *Desmidorchis penicillata*, *D. retrospiciens* [80], *M. quadrangula* (syn. *Caralluma quadrangula*) [78], *Ceropegia sunhangiana* [112], *C. longifolia*, *C. nilotica*, *C. dolichophylla*, *Huernia keniensis*, and *O. variegata* [75].

Across both *Orbea* species, the IR/LSC boundaries were found to be more conserved than the IR/SSC boundaries, a pattern that is consistent with other Apocynaceae plastomes [75,78,80]. The evolutionary significance of IR boundary variation lies in its impact on plastome structure and gene content. Expansion of IRs can lead to gene duplications, as demonstrated in *Pelargonium* [113], while extreme contraction or complete loss of IRs—as observed in some Fabaceae species like *Cicer arietinum* [114] and in *Passiflora* (Passifloraceae) [115]—can result in gene loss and increased genome instability. Such structural changes can have downstream effects on genome stability, recombination frequency, and even evolutionary rates of genes [116]. Therefore, comparative analysis of IR boundaries offers valuable insight into the mechanisms of chloroplast genome evolution and the lineage-specific structural adaptations of plant plastomes.

Analyzing the intron and exon content of chloroplast genomes provides critical insights into genome evolution, RNA splicing mechanisms, and structural variations, and can yield valuable phylogenetic markers (e.g., [117,118]). In this study, examination of *O. sprengeri* subsp. *commutata* and *O. wissmannii* var. *eremastrum* revealed the presence of 17 unique intron-containing genes in each plastome, predominantly distributed across LSC and IR regions. This gene count and distribution pattern are consistent with those reported in diverse angiosperm lineages (e.g., [48,119,120]. Furthermore, several highly conserved features typical of land plant chloroplasts were observed, such as the presence of two introns within both *ycf3* and *clpP1*, and the characteristically large intron (2527–2530 bp) associated with the *trnK-UUU* gene [109,121,122,123].

Codon usage analysis is a valuable tool for understanding the molecular evolution, translational efficiency, and nucleotide composition bias of plastid genomes. Each amino acid in a protein sequence may be encoded by one (as in methionine and tryptophan) to six synonymous codons, and the usage frequency of these codons can differ across species—and even among genes within the same organism [124,125]. In chloroplasts, codon usage is shaped by both mutational pressure and natural selection, and it can influence gene expression levels, protein structure, and the efficiency of translation [126,127,128]. In this study, codon usage and amino acid composition in *O. sprengeri* subsp. *commutata* and *O. wissmannii* var. *eremastrum*, along with other Ceropegieae plastomes compared, exhibited strong conservation. Preferred codons were predominantly A/U-ending, reflecting an AT-rich bias in the third codon position that is characteristic of most land plant chloroplast genomes [83,121,122,129]. This codon bias was mirrored in amino acid composition, with leucine being the most abundant (~10.5–10.9%), followed by isoleucine and serine, which are crucial for chloroplast-encoded proteins involved in photosynthesis and gene regulation [130]. Less frequent usage of codons encoding tryptophan and cysteine is consistent with their limited codon representation and functional specificity. These patterns align with findings in other species of Apocynaceae and angiosperms [78,80,121,122,131].

Mutational hotspots in chloroplast genomes—regions with elevated nucleotide diversity—are invaluable for developing DNA barcodes and resolving phylogenetic relationships [132]. In this study, six loci—*accD*, *clpP*, *ndhF*, *ycf1*, *psbM–trnD*, and *rpl32–trnL*—were identified as the most variable regions (Pi = 0.048–0.076) across Ceropegieae plastomes. These loci correspond to hotspot regions reported in other Apocynaceae plastomes [75,78,80]. While several studies have evaluated the discriminating power of conventional plastid markers in Apocynaceae (e.g., *matK*, *rbcL*, *trnH–psbA*, *trnL–trnF*) [133,134,135,136,137,138,139,140], the six highly variable loci identified here have not previously been evaluated as DNA barcodes. Traditional barcodes such as *matK* and *rbcL* exhibited low variability (Pi < 0.0025 and 0.0017, respectively), indicating limited discriminatory power for Ceropegieae. Similarly, the widely used *trnL* intron and *trnL–trnF* spacer—commonly applied in Ceropegieae phylogenetic studies [4,5,6,141,142,143,144,145]—show only moderate divergence (Pi ≤ 0.032), consistent with the findings of Alharbi [135], who also reported their limited resolution among closely related taxa.

Among the six hotspots, *ycf1* and *ndhF* stand out: both have been proposed as superior plastid barcodes in diverse angiosperm lineages [146,147] and offer phylogenetic insight and species-level discrimination for applications in breeding [148,149,150]. *AccD*, encoding the acetyl-CoA carboxylase subunit D [151], has aided species delimitation in genera like *Hexachlamys* [152] and *Chamaecyparis* [153], although its use can be complicated by mitochondrial or nuclear paralogs [154]. Likewise, *clpP*, a plastid protease subunit, evolves rapidly in certain angiosperm lineages [155] and has been used as a barcode in Actinidiaceae [153], *Dracaena* [156], and *Prunus* [157], though its long sequence length can pose practical amplification challenges [156].

The intergenic spacers *psbM–trnD* and *rpl32–trnL* have also demonstrated high species-resolution capacity in various taxa, including *Panax* [158], *Astragalus* [159], *Acacia* [160], and *Leptochloa* [161], especially when used in combination with other markers. However, these regions often require careful alignment due to high sequence divergence and potential structural variation. Collectively, these six highly variable loci reflect a broader evolutionary pattern in which elevated substitution rates are localized to specific plastid regions rather than occurring uniformly across the genome [155]. Recent syntheses of plant organellar genome evolution emphasize that such localized mutation hotspots arise from the interaction of DNA repair mechanisms, functional constraints, and selection, rather than from genome-wide increases in mutation rates [162]. In this context, the candidate loci identified in this study lay the groundwork for future targeted DNA barcode validation research within the tribe Ceropegieae, particularly among morphologically challenging and closely related species.

Simple sequence repeats (SSRs), also known as microsatellites, consist of short DNA motifs (1–6 bp) that occur in tandem and are widely applied in DNA barcoding, population-level analyses, and phylogenetic research because they are highly variable and typically codominant [163,164,165]. In chloroplast genomes, SSR loci generally originate from replication slippage or errors that arise during repair or recombination events [166]. The composition and distribution of these repeats can provide insights into evolutionary patterns and may distinguish lineages or genera [167,168,169]. In the present analysis, mononucleotide SSRs were the most common repeat class detected across all plastomes, with A/T motifs being the most prevalent—a trend consistent with earlier reports from land plants [121,122,164,170,171].

Dispersed repetitive DNA sequences, another important class of plastid genome repeats, were also identified and are known to play a significant role in genome rearrangements and structural variation [172,173]. In addition, they may influence nucleotide substitution rates, thereby contributing to the evolutionary dynamics of the plastome [174]. *Orbea wissmannii* var. *eremastrum* exhibited a species-specific repeat profile, characterized by a higher number of reverse repeats (n = 18) and the presence of five complemented repeats, which were completely absent in *O. sprengeri* subsp. *commutata* and other closely related Stapeliinae taxa. This distinct repeat architecture distinguishes *O. wissmannii* var. *eremastrum* from its relatives, where forward and palindromic repeats are typically more abundant. In all examined species, most repeats were concentrated in IGS regions—a distribution pattern commonly observed in previous plastome studies [175,176]. These findings underscore the potential of SSRs and dispersed repeats as valuable resources for assessing genetic diversity and evolutionary divergence within *Orbea* species.

The Ka/Ks ratio (also referred to as dN/dS or ω) is a key metric used to infer the nature and strength of selective pressures on protein-coding genes. A Ka/Ks ratio <1 indicates purifying selection, where deleterious amino acid changes are eliminated, maintaining functional conservation. A ratio of 1 suggests neutral evolution, while values >1 indicate positive selection, where beneficial mutations are favored [177,178,179]. Analysis of 79 protein-coding genes across 15 Ceropegieae plastomes revealed that most genes are under strong purifying selection, reflecting their essential roles in plastid function. Notably, no genes were found to be evolving neutrally. However, four genes—*accD*, *ndhE*, *ycf1*, *and ycf2*—exhibited Ka/Ks ratios >1, indicating signals consistent with positive selection. The potential functional and adaptive significance of these genes is discussed below in a hypothesis-driven context, in relation to their known roles in plastid metabolism and stress adaptation.

The *accD*, which encodes a subunit of the plastid acetyl-CoA carboxylase complex critical for fatty acid biosynthesis [180], showed evidence of accelerated evolution. This gene has also been reported to evolve rapidly in *Medicago ruthenica* [181], *Ficus* [182], and cupressophytes [180], often due to repetitive sequence insertions that contribute to both high substitution rates and genome rearrangements. In Ceropegieae, many of which are succulents inhabiting arid environments, such selection on *accD* may reflect adaptive modifications in lipid metabolism that enhance membrane stability, cuticular wax formation, or water retention.

Similarly, *ndhE*, part of the NADH dehydrogenase complex involved in cyclic electron flow and chlororespiration [89], exhibited Ka/Ks patterns consistent with positive selection. This may suggest potential fine-tuning of photosynthetic energy balance under stress conditions such as drought or fluctuating light, consistent with the ecological niches of Ceropegieae. Unlike its loss in some non-photosynthetic plants [84], selection on *ndhE* here may indicate adaptive maintenance associated with stress resilience.

*ycf1*, one of the largest and most variable chloroplast genes, although its precise function has been a subject of debate, has been identified as Tic214, a vital component of the *Arabidopsis* TIC (Translocon at the Inner Chloroplast envelope) complex [146]. Despite its conserved function, *ycf1* displays high sequence variability and has been identified as putatively positively selected in *Ficus* [182], *Caragana* [183], and *Medicago ruthenica* [181]. In Ceropegieae, elevated Ka/Ks values for *ycf1* may reflect adaptive changes in plastid protein trafficking, potentially enhancing import specificity or efficiency under environmental stress.

*ycf2*, the largest plastid gene in angiosperms, is unequivocally identified as a functional gene whose products are essential for cell survival and plant viability [184], and also showed signatures consistent with adaptive evolution. Similar selection has been documented in *Cerasus* [185] and Zingiberaceae [186], where it is associated with adaptations to varying light conditions. In Ceropegieae, elevated Ka/Ks values for *ycf2* may be associated with optimization of ATP-driven protein transport in response to the energy demands of plastid function under arid or fluctuating light conditions. Collectively, while most plastid genes remain under purifying selection, *accD*, *ndhE*, *ycf1*, and *ycf2* may contribute to evolutionary flexibility, enabling persistence in diverse and challenging habitats.

Recent advances in phylogenomics—driven by the increasing accessibility of high-throughput sequencing—have substantially improved the resolution of plant evolutionary relationships compared with earlier approaches based on one or a few loci [187,188]. During the Sanger sequencing era, commonly used markers such as *rbcL* and *matK* provided broad phylogenetic frameworks (e.g., [189,190], culminating in the Angiosperm Phylogeny Group classification [191]. However, phylogenies inferred from a limited number of loci often yielded weakly supported or conflicting topologies due to rate heterogeneity and locus-specific evolutionary histories [192,193,194]. Plastome-scale datasets, owing to their conserved structure, compact genome size, and relatively low recombination rates [195,196], represent a powerful resource for plastid-based phylogenomic inference and hypothesis generation across diverse plant lineages [188]. Nevertheless, because plastid genomes constitute a single, typically maternally inherited locus [197,198], plastome-based phylogenies may be influenced by processes such as chloroplast capture, introgression, or incomplete lineage sorting [199,200,201], and should therefore be interpreted as reflecting plastid evolutionary histories rather than definitive species relationships.

In the present study, phylogenomic analysis of 21 plastomes produced a well-resolved plastid phylogeny of tribe Ceropegieae, with strong support for major nodes (Figure 10). The inferred relationships among subtribes are broadly congruent with previous hypotheses based on limited gene regions [4,5], but with significantly improved resolution. Within this plastid framework, *Orbea* taxa were placed within a well-supported clade corresponding to the stem-succulent stapeliads of subtribe Stapeliinae. However, the three *Orbea* plastomes did not form a monophyletic group: the newly sequenced Arabian taxa (*O. sprengeri* subsp. *commutata* and *O. wissmannii* var. *eremastrum*) were recovered as sister lineages, whereas *O. variegata* from South Africa was resolved in a separate clade with *Stapelia gigantea*. This pattern is consistent with earlier multilocus analyses indicating that relationships among *Orbea* species may be more closely associated with geographic structure than with floral morphology [6]. Similar biogeographic structuring has been reported in other succulent lineages, including *Huernia* [6], *Erica* L. [202], and *Euphorbia* subg. *Athymalus* [203]. While such patterns may reflect historical diversification across distinct ecological regions, these interpretations should be regarded as hypotheses that require explicit testing using nuclear genomic data and functional approaches.

From a conservation perspective, the plastome data generated here provide immediately applicable genomic resources for species identification, phylogenetic placement, and the development of informative plastid markers for *Orbea*, particularly endemic and threatened Arabian taxa. These data may support conservation planning by facilitating accurate taxonomic delimitation, identifying evolutionarily distinct lineages, and informing the selection of priority taxa for further genetic study in the Arabian Peninsula. In the longer term, integration of plastome data with nuclear genomic datasets, population-level sampling, and ecological information will be essential to refine species boundaries, assess gene flow, and robustly evaluate adaptive hypotheses across *Orbea* and allied Ceropegieae lineages.

## 5. Conclusions

This study provides the first complete chloroplast genomes of two Arabian *Orbea* taxa—*O. sprengeri* subsp. *commutata* and *O. wissmannii* var. *eremastrum*—and offers new insights into plastome structure, variation, and evolutionary placement of these taxa within the tribe Ceropegieae. Despite the limited taxon sampling within *Orbea* (only three plastomes out of over 60 described species), the findings reveal unique structural features specifically in the Arabian taxon *O. wissmannii* var. *eremastrum*, including a flip-flop inversion spanning *ndhG* to *ndhF* and extreme IR expansion, which contribute to plastome size variation, gene rearrangements, and gene duplication. The observed structural divergence in this taxon may be a sign of plastome heteroplasmy, prompting further investigation through broader population-level sampling. Although the current data set does not allow for broad generalizations across the genus *Orbea*, the results highlight the genomic distinctiveness of Arabian *Orbea* taxa analyzed here relative to the only previously sequenced African representative (*O. variegata*). Expanding plastome samples across *Orbea* is essential to capture its full genomic diversity and to clarify evolutionary trends within Ceropegieae. Within this context, the present study lays a foundation for future plastid phylogenomic, DNA barcoding, and conservation research of arid-adapted Arabian *Orbea* taxa.

## Figures and Tables

**Table 1:** List of endemic *Orbea* taxa recorded from the Arabian Peninsula.

	Species	Distribution
1	*Orbea araysiana* (Lavranos & Bilaidi) Bruyns	Yemen
2	*Orbea chrysostephana* (Deflers) Bruyns	Yemen
3	*Orbea cucullata* (Plowes) Meve	Yemen
4	*Orbea deflersiana* (Lavranos) Bruyns	Yemen, Oman, Saudi Arabia
5	*Orbea fenestrata* (Plowes) Meve	Yemen
6	*Orbea luntii* (N.E.Br.) Bruyns	Yemen, Oman
7	*Orbea nardii* Raffaelli, Mosti & Tardelli	Oman
8	*Orbea parviloba* (Bruyns) Meve	Yemen, Oman
9	*Orbea sprengeri* subsp. *commutata* (A. Berger) Bruyns	Yemen, Oman, Saudi Arabia
10	*Orbea wissmannii* (O. Schwartz) Bruyns	Yemen, Oman, Saudi Arabia
11	*Orbea wissmannii* var. *wissmannii*	Yemen, Oman, Saudi Arabia
12	*Orbea wissmannii* var. *eremastrum* (O. Schwartz) Bruyns	Yemen, Oman, Saudi Arabia

**Table 2:** List of available chloroplast genomes from the tribe Ceropegieae used in this study. Species included only in the phylogenetic analysis (due to partial plastome sequences) are highlighted in light gray. The two plastomes newly generated in this study are indicated in bold.

Tribe Subtribe	Species	GenBank Accession No.
Ceropegieae		
Anisotominae	*Sisyranthus trichostomus* (Harv.) K.Schum.	KF539852
Heterostemminae	*Heterostemma oblongifolium* Costantin	OP133585
Leptadeniinae	*Pentasachme caudatum* Wall. ex Wight	MW136321
	*Leptadenia albida* (Schinz) Bruyns	MG963261
	*Leptadenia pyrotechnica* (Forssk.) Decne.	MW546533
Stapeliinae	*Ceropegia dolichophylla* Schltr.	OP133580
	*Ceropegia longifolia* Wall.	OP133586
	*Ceropegia nilotica* Kotschy	OP133584
	*Ceropegia sunhangiana* P.R.Luo & T.Deng	OR260539
	*Desmidorchis penicillata* (Deflers) Plowes	PP101941
	*Desmidorchis retrospiciens* Ehrenb.	PP291741
	*Duvalia velutina* Lavranos	MT431578
	*Huernia keniensis* R.E.Fr.	OP133582
	*Monolluma quadrangula* (Forssk.) Plowes	MT413385
	*Orbea variegata* (L.) Haw.	NC_079601
	***Orbea sprengeri*** **subsp. *commutata* (A. Berger) Bruyns**	**PQ412530**
	***Orbea wissmannii*** **var. *eremastrum* (O. Schwartz) Bruyns**	**PQ412531**
	*Stapelia gigantea* N.E.Br.	MG963259

**Table 3:** Gene composition encoded by the chloroplast genomes of the of *Orbea sprengeri* subsp. *commutata* and *Orbea wissmannii* var. *eremastrum.* Genes shown in **bold text** indicate duplications found exclusively in *O. wissmannii* var. *eremastrum*.

Category	Group of Genes	Name of Genes	Number
Self-replication	Ribosomal RNA genes (rRNA)	*rrn5* ^(×2)^, *rrn4.5* ^(×2)^, *rrn16* ^(×2)^, *rrn23* ^(×2)^	8 (4)
	Transfer RNA genes (tRNA)	*trnA-UGC*^+ (×2)^, *trnC-GCA*, *trnD-GUC*, *trnE-UUC*, *trnF-GAA*, *trnfM-CAU*, *trnG-GCC*, *trnG-UCC*, *trnH-GUG*, *trnI-CAU* ^(×2)^, *trnI-GAU*^+, (×2)^, *trnK-UUU*^+^, *trnL-CAA* ^(×2)^, *trnL-UAA*^+^, ***trnL-UAG* ^(×2)^**,* trnM-CAU*, *trnN-GUU* ^(×2)^, *trnP-UGG*, *trnQ-UUG*, *trnR-ACG* ^(×2)^, *trnR-UCU*, *trnS-GCU*, *trnS-GGA*, *trnS-UGA*, *trnT-GGU*, *trnT-UGU*, *trnV-GAC* ^(×2)^, *trnV-UAC*^+^, *trnW-CCA*, *trnY-GUA*	37 (7) **38 (8)**
	Small subunit of ribosome	*rps2*, *rps3*, *rps4*, *rps7* ^(×2)^, *rps8*, *rps11*, *rps12*^+, (×2)^, *rps14*, ***rps15** ***^(×2)^**, *rps16*^+^, *rps18*, *rps19*	14 (2) **15 (3)**
	Large subunit of ribosome	*rpl2*^+, (×2)^, *rpl14*, *rpl16*^+^, *rpl20*, *rpl22*, *rpl23* ^(×2)^, ***rpl32** ***^(×2)^**, *rpl33*, *rpl36*	11 (2) **12 (3)**
	DNA-dependent RNA polymerase	*rpoA*, *rpoB*, *rpoC1*^+^, *rpoC2*	4
Genes for photosynthesis	Photosystem I	*psaA psaB psaC psaI*, *psaJ*, *ycf3*,^++^ *ycf4*	7
	Photosystem II	*psbA*, *psbB*, *psbC*, *psbD*, *psbE*, *psbF*, *psbH*, *psbI*, *psbJ*, *psbK*, *psbL*, *psbM*, *psbN*, *psbT*, *psbZ*	15
	Subunits of cytochrome b/f complex	*petA*, *petB*^+^, *petD*^+^, *petG*, *petL*, *petN*	6
	Subunits of ATP synthase	*atpA*, *atpB*, *atpE*, *atpF*^+^, *atpH*, *atpI*	6
	Large subunit of rubisco	*rbcL*	1
	Subunits of NADH-dehydrogenase	***ndhA***^+^ ^**(×2)**^, *ndhB*^+, (×2)^, *ndhC*, *ndhD*, *ndhE*, ***ndhF* ^(×2)^**, *ndhG*, ***ndhH* ^(×2)^**, ***ndhI* ^(×2)^**, *ndhJ*, *ndhK*	12 (1) **16 (5)**
Other genes	Chloroplast envelope membrane protein	*cemA*	1
	Maturase	*matK*	1
	ATP-dependent protease subunit P	*clpP* ^++^	1
	Subunit acetyl-coA carboxylase	*accD*	1
	C-type cytochrome synthesis	*ccsA*	1
	Translational initiation factor	*infA*	1
	Hypothetical proteins	*ycf2* ^(×2)^, *ycf15* ^(×2)^	4 (2)
	Component of TIC complex	*ycf1* ^(×2)^	2 (1)
Total		*O. sprengeri* subsp. *commutate* 133 (19) *O. wissmannii* var. *eremastrum* **140 (26)**	

+ Gene containing one intron; ++ gene containing two introns; (×2) indicates that the number of the repeat unit is 2; (×2) duplicated only in *O. wissmannii* var. *eremastrum;* number in parentheses indicates the number of duplicated genes.

**Table 4:** Lengths of introns and exons in the split genes of the chloroplast genomes of *Orbea sprengeri* subsp. *commutata* and *Orbea wissmannii* var. *eremastrum*.

Gene	Location	Start		End		Exon I		Intron I		Exon II		Intron II		Exon III	
		*O. sprengeri*	*O. wissmannii*	*O. sprengeri*	*O. wissmannii*	*O. sprengeri*	*O. wissmannii*	*O. sprengeri*	*O. wissmannii*	*O. sprengeri*	*O. wissmannii*	*O. sprengeri*	*O. wissmannii*	*O. sprengeri*	*O. wissmannii*
*trnK-UUU*	LSC	1729	1792	4331	4390	37	37	2530	2527	36	35				
*rps16*	LSC	5132	5182	6223	6273	40	40	825	825	227	227				
*trnG-UCC*	LSC	9199	9264	9936	10,001	23	23	667	667	48	48				
*atpF*	LSC	12,002	12,002	13,253	13,254	145	145	700	701	407	407				
*rpoC1*	LSC	21,158	21,136	23,973	23,959	432	432	779	787	1605	1605				
*ycf3*	LSC	44,325	44,601	46,355	46,684	124	124	744	797	230	230	780	780	153	153
*trnL-UAA*	LSC	49,118	49,441	49,684	50,007	35	35	482	482	50	50				
*trnV-UAC*	LSC	53,797	54,114	54,456	54,773	38	38	587	587	35	35				
*petB*	LSC	77,135	77,418	78,529	78,956	6	6	747	891	642	642				
*petD*	LSC	78,706	79,133	80,004	80,431	8	8	810	810	481	481				
*rpl16*	LSC	83,503	83,940	84,857	85,294	9	9	935	935	411	411				
*clpP1*	LSC	72,161	72,357	74,232	74,515	71	71	749	749	292	292	732	815	228	228
*rpl2*	IR	86,523	86,955	87,993	88,425	391	391	646	646	434	434				
*ndhB*	IR	96,861	97,215	99,077	99,431	775	775	684	684	758	758				
*trnI-GAU*	IR	104,369	104,723	105,388	105,742	37	37	948	948	35	35				
*trnA-UGC*	IR	105,452	105,806	106,341	106,695	38	38	817	817	35	35				
*ndhA*	SSC(IR)	127,200	127,302	129,405	129,507	553	553	1108	1108	545	545				

(IR) indicates that *ndhA* in *O. wissmannii* var. *eremastrum* is located within the inverted repeat region.

**Table 5:** Comparison of chloroplast genome features of tribe Ceropegieae species. Subtribe Stapeliinae (*O* = *Orbea*, *Hu* = *Huernia*, *Du* = *Duvalia*, *M* = *Monolluma*, *D* = *Desmidorchis*, *C* = *Ceropegia*), subtribe Heterostemminae (*H* = *Heterostemma*), and subtribe Leptadeniinae (*L* = *Leptadenia*, *P* = *Pentasachme*).

	Species														
Features	*O. sprengeri* subsp. *commutata*	*O. wissmannii* var. *eremastrum*	*O. variegata*	*Hu. keniensis*	*Du. velutina*	*M. quadrangula*	*D. penicillata*	*D. retrospiciens*	*C. dolichophylla*	*C. nilotica*	*C. longifolia*	*C. sunhangiana*	*H. oblongifolium*	*L. pyrotechnica*	*P. caudatum*
Genome size (bp)	162,017	170,054	161,348	159,812	161,018	161,456	161,776	161,861	161,201	160,961	161,223	161,376	162,509	161,510	158,487
LSC length (bp)	86,501	85,882	85,886	85,024	85,728	85,992	86,107	85,990	85,966	85,911	85,987	86,153	90,380	89,590	91,022
SSC length (bp)	13,213	4298	13,044	13,306	13,085	13,38	13,273	13,264	13,339	13,228	13,340	13,363	18,585	20,026	19,834
IR length (bp)	31,151	39,937	31,209	30,741	31,103	31,042	31,198	31,303	30,948	30,911	30,948	30,930	24,761	25,947	24,057
Number of genes	133	140	132	131	133	129	133	133	131	131	131	131	127	133	127
Number of protein-coding genes	88	94	87	86	88	85	88	88	86	86	86	86	83	88	83
Number of tRNA genes	37	38	37	37	37	36	37	37	37	37	37	37	36	37	36
Number of rRNA genes	8	8	8	8	8	8	8	8	8	8	8	8	8	8	8
Total GC content (%)	37.7	37.4	37.9	37.9	37.9	38	37.8	37.7	37.9	37.9	37.8	37.8	37.8	37.4	37.8
GC content in LSC (%)	36.1	36.1	36.2	36.3	36.3	36.2	36.1	36.17	36.2	36.2	36.2	36.2	36	35.4	36
GC content in SSC (%)	32.9	34.2	33	32.7	32.8	32.8	32.8	32.31	32.8	33	32.8	32.8	32.4	31.8	32.4
GC content in IR (%)	40.8	38.9	41.1	41.3	41.1	41.2	41	43.35	41.2	41.3	41.2	41.2	43.2	43	43.2
Coding sequences (bp)	85,020	90,876	85,767	85,128	85,519	74,505	85,494	85,611	85,434	85,371	85,482	85,404	82,098	81,522	82,098
Percentage of coding sequences (%)	52.4	53.1	53.15	53	53	46	53	53	53	53	53	53	51	50	52
Non-coding sequences (bp)	76,997	79,178	75,581	74,684	75,499	86,951	76,282	76,666	75,767	75,590	75,741	75,972	80,411	79,988	76,389

## Data Availability

The data generated in this study are available in the article and Supplementary Material. The whole chloroplast genome sequence of *Orbea sprengeri* subsp. *commutata and Orbea wissmannii* var. *eremastrum* is available for download from GenBank at https://www.ncbi.nlm.nih.gov/ (accession NO.: PQ412530 and PQ412531, respectively).

## Supplementary Materials

The following supporting information can be downloaded at: https://www.mdpi.com/article/10.3390/biology15030223/s1, Figure S1. Sliding window analysis plots of nucleotide diversity (Pi) across the complete chloroplast genomes: (A) among the 15 Ceropegieae taxa and (B) within the three *Orbea* taxa; Figure S2. The frequency of SSR motifs in chloroplast genomes of tribe Ceropegieae species; Figure S3. Distribution of SSRs across different plastome regions (LSC, IRA, SSC, IRB) in three *Orbea* taxa; Figure S4: Dispersed repeats number in the different regions of Ceropegieae chloroplast genomes; Table S1: Tracing the taxonomic history of *Orbea* species recorded in the Arabian Peninsula, spanning the period from 1896 to 2017; Table S2: Putative preferred codons in the Ceropegieae chloroplast genomes; Table S3: Distribution to different simple sequence repeats (SSRs) type classes; Table S4: Frequency of classified simple sequence repeats (SSRs) motifs; Table S5: Dispersed repeat sequences present in the chloroplast genomes of Ceropegieae species; Table S6: Ka/Ks ratio analysis of 79 protein-coding genes across 15 chloroplast genomes of tribe Ceropegieae.

## Abbreviations

The following abbreviations are used in this manuscript:

RSCU	Relative synonymous codon usage
LSC	Large single-copy
SSC	Small single-copy
IR	Inverted repeat
IGS	Intergenic spacer
Pi	Nucleotide diversity
SSRs	Simple sequence repeats
CDS	Coding sequences
Ka	Non-synonymous substitution rate
Ks	Synonymous substitution rate
Ka/Ks	Nonsynonymous to synonymous substitution rate ratios
ML	Maximum likelihood
BI	Bayesian inference
ESS	Effective sample sizes
PP	Posterior probabilities
