# Supplementary material for: First Plastome Sequences of Two Endemic Taxa of Orbea Haw. from the Arabian Peninsula: Comparative Genomics and Phylogenetic Relationships Within the Tribe Ceropegieae (Asclepiadoideae, Apocynaceae)
Source: Biology (Basel). 2026 Jan 25;15(3):223. doi: 10.3390/biology15030223 (PMC12896904; doi:10.3390/biology15030223)
Supplement: Supplementary file 1 [file biology-15-00223-s001.zip › Figures S1,S2,S3,S4.pdf]

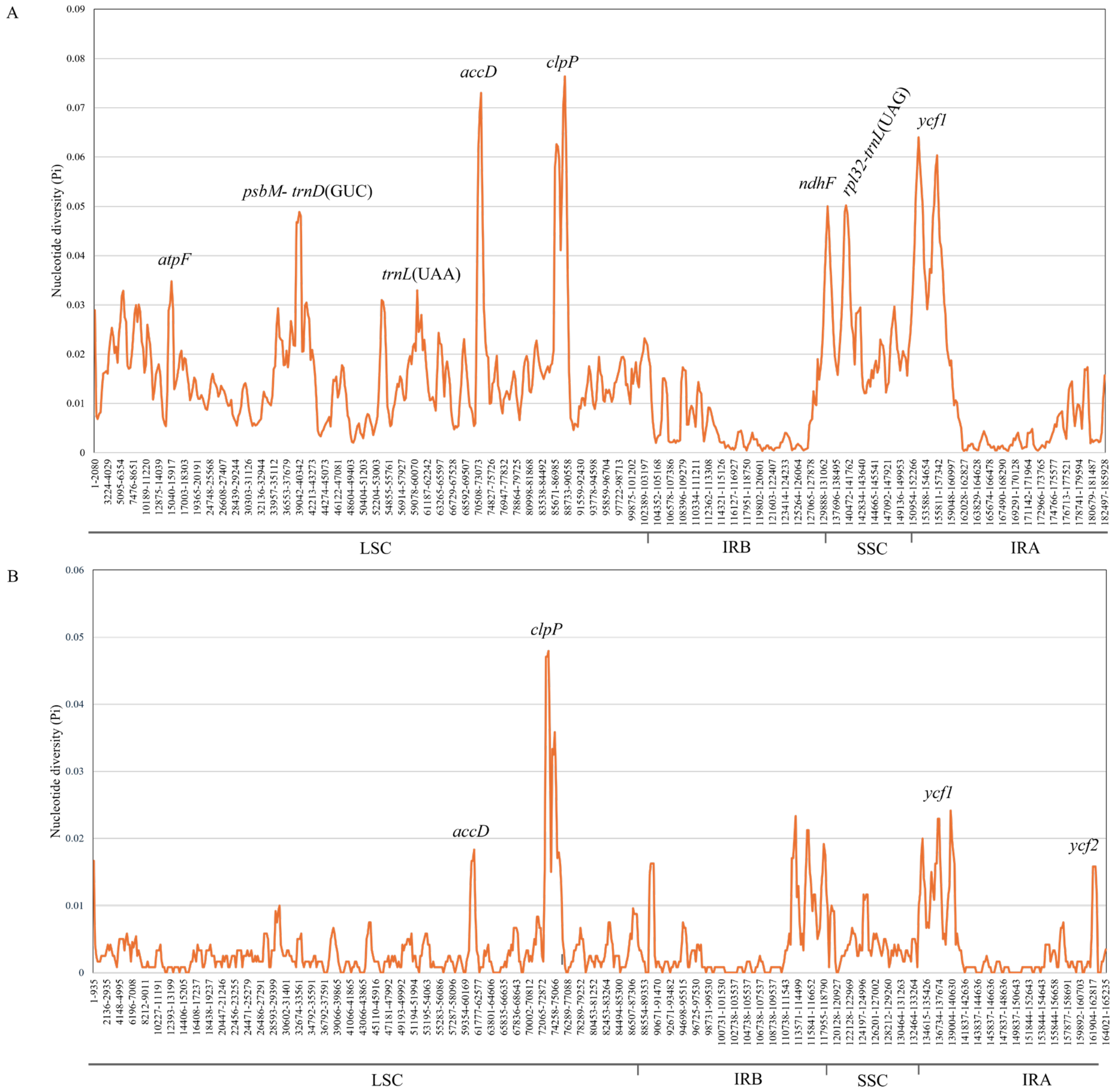


Figure S1. Sliding window analysis plots of nucleotide diversity (Pi) across the complete chloroplast genomes: (A) among the 15 Ceropegieae taxa and (B) within the three *Orbea* taxa (*Orbea wissmannii* var. *eremastrum*, *O. sprengeri* subsp. *commutata*, and *O. variegata*). Window length: 800 bp; Step size: 200 bp.

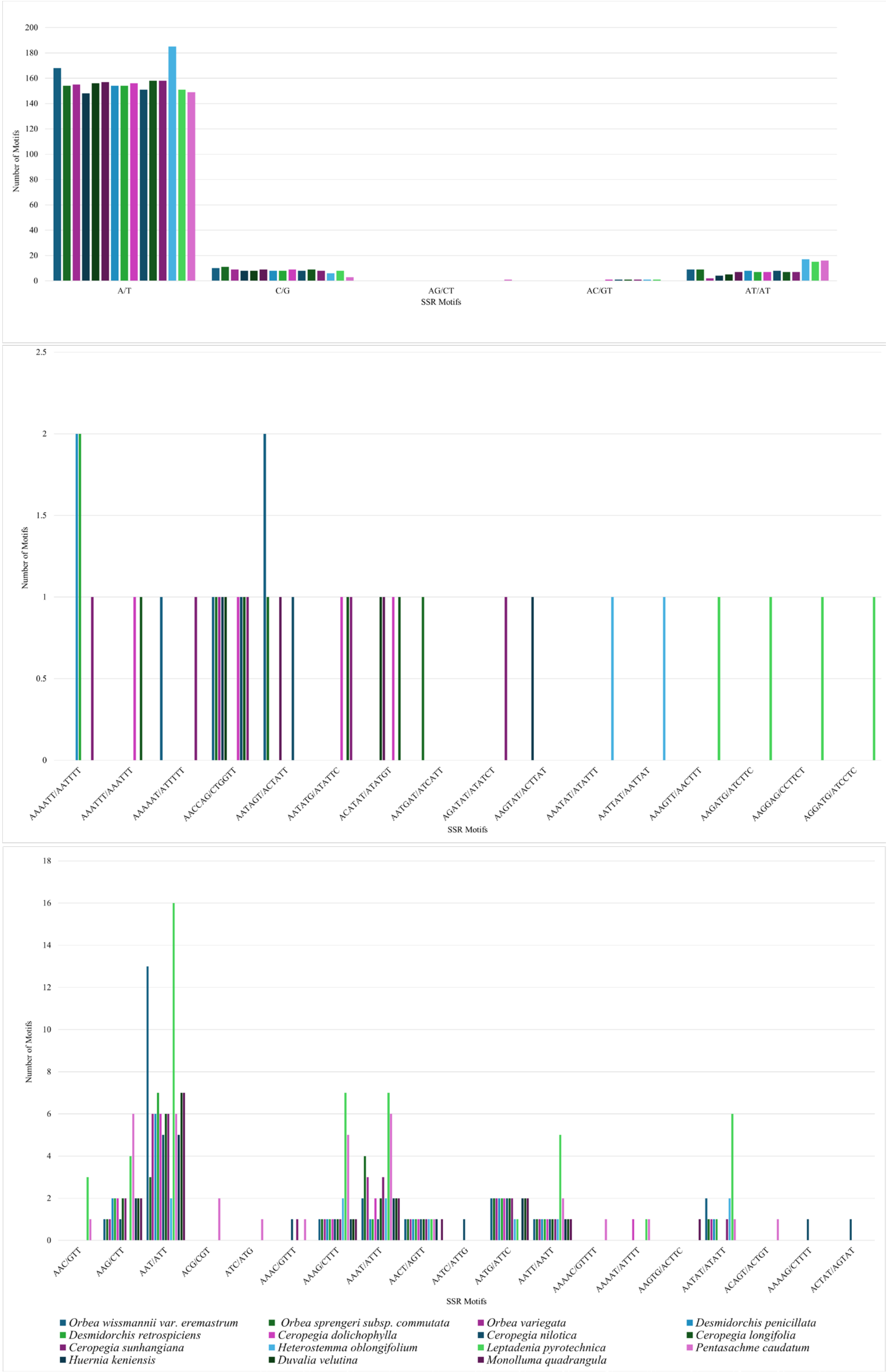


Figure S2. The frequency of SSR motifs in chloroplast genomes of tribe Ceropegieae species.

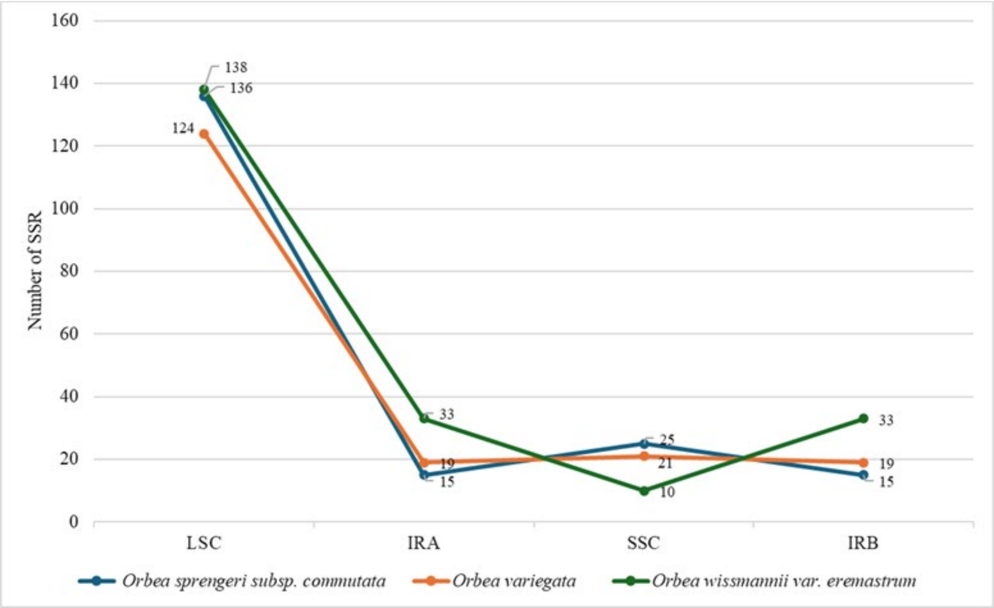


Figure S3. Distribution of SSRs across different plastome regions (LSC, IRA, SSC, IRB) in three *Orbea* taxa.

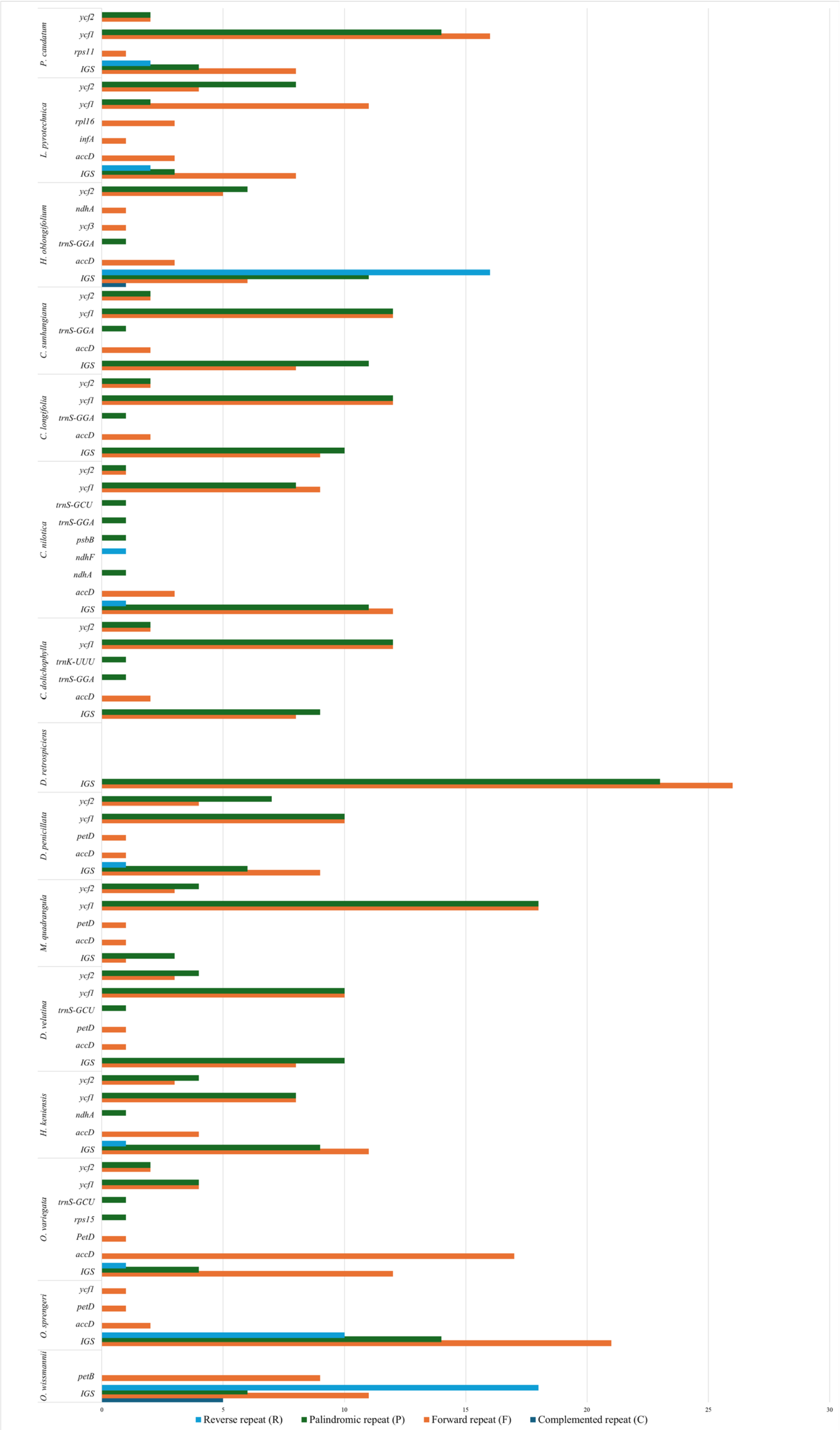


Figure S4. Dispersed repeats number in the different regions of Ceropegieae chloroplast genomes
